# Efficacy of Esketamine nasal spray in treatment-resistant depression: impact on depressive symptoms,hopelessness and suicide risk: A real-world evidence study

**DOI:** 10.1192/j.eurpsy.2025.684

**Published:** 2025-08-26

**Authors:** M. Pompili, M. A. Trocchia, L. Longhini, E. Dispenza, C. Di Legge, S. Sarubbi, D. Erbuto, I. Berardelli

**Affiliations:** 1 Dept. of Neurosciences, Mental Health and Sensory Organs, Sapienza University of Rome; 2 Psychiatry Residency Training Program, Faculty of Medicine and Psychology, Sapienza University of Rome, Psychiatry Unit, Sant’Andrea Hospital; 3 Department of Human Neurosciences, Sapienza University of Rome, Viale dell’Università, Rome, Italy

## Abstract

**Introduction:**

Treatment-resistant depression (TRD) is a condition associated with multiple severe implications and high suicide risk. Esketamine is a pharmacological agent approved by the FDA and EMA recommended for adults diagnosed with TRD. The glutamatergic mechanisms of depression are currently the subject of numerous studies. Esketamine has glutamatergic neuromodulatory properties to enhance the effects of selective serotonin and norepinephrine reuptake inhibitors. Furthermore, the anti-suicidal effect of esketamine represents a reason to explore its properties since managing suicide risk in patients with MDD is complex.

**Objectives:**

The present observational, retrospective study aimed to assess the efficacy and tolerability of Esketamine in a real-world outpatient setting. A secondary objective was to explore the potential benefits on hopelessness and suicide risk(suicide ideation and suicide attempts) to establish future research directions that may enrich knowledge on the effects of esketamine on suicide risk in patients with resistant depression.

**Methods:**

20 patients diagnosed with treatment-resistant depression disorder were treated with Esketamine.Depressive symptoms (MADRS and BDI), suicide risk(C-SSRS), and hopelessness(BHS)were assessed. Repeated measures analyses were conducted to evaluate changes from baseline(T0) and 3-month follow-up(T1).

**Results:**

11 patients(60%)were females, and the average age of the sample was 49.40 (SD= 17.38). 9 patients(45%)were single,and 11 patients (55%) were employees.At baseline,11 patients(55%)reported suicidal ideation,and 2 patients(10%)attempted suicide in the previous 3 months. Of the 20 patients, 4 patients did not complete the treatment and were excluded from analyses. Results showed that depressive symptoms decreased at the end of treatment (t1) compared to baseline (t0).Specifically,both clinician (t=7.32;p<.001) and self-report (t=4.34;p<.001)measures show lower levels of depressive symptoms(MADRS t0=33.31±4.35vs.t1= 20.13±5.31,and BDI t0=42.13±7.97 vs.t1=29.31±9.96, respectively).About suicide risk, at baseline 11 patients reported some level of suicidal ideation while at (T1) 7 patients did; the difference was not statistically significant(p=.125);conversely,a significant decrease in suicidal ideation intensity was found (t=2.48,p<.05; t0=5.37±5.77vs.t1=1.87±3.51). Finally,patients also reported decreased hopelessness levels(BHS;t=3.97,p=.001; t0=16.19±3.12 vs. t1=12.12±4.27).

**Image 1:**

**Figure:**
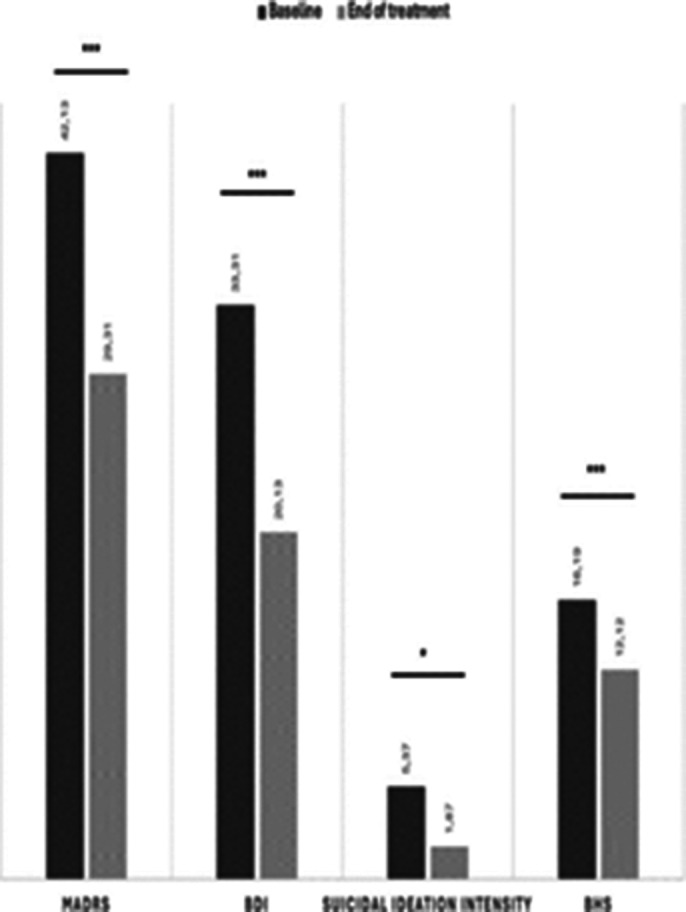

**Conclusions:**

Our findings demonstrated a robust overall response regarding depressive symptoms after Esketamine in TRD. A notable response concerning suicide ideation intensity was also observed in patients with resistant depression; however, further studies are necessary to better investigate the role of esketamine in suicide prevention.

**Disclosure of Interest:**

M. Pompili Consultant of: M Pompili wishes to disclose that in the last five years, he has received lectures and advisory board honoraria or has engaged in clinical trial activities with Angelini Pharma, Allergan, Janssen, Lundbeck, Merck Sharp and Dohme, Otsuka, Rovi, Pfizer, Fidia, Viatris, Recordati,Boehringer Ingelheim and Teva, M. A. Trocchia: None Declared, L. Longhini: None Declared, E. Dispenza: None Declared, C. Di Legge: None Declared, S. Sarubbi: None Declared, D. Erbuto: None Declared, I. Berardelli: None Declared

